# 

**Published:** 1883-08-20

**Authors:** 


### *EDITORIAL NOTICES.*

COLLEGE ADVERTISEMENTS.—Read the College advertisements.

TONGALINE.—See the advertisement of this article in this number of our Journal; especially adapted to neuralgia and rheumatism.

FRED STEARNS & CO.—See the advertisement of this great drug establishment of Detroit, Michigan. The house is old and well established, and their preparations neat, varied, beautiful and reliable.

SCHOOL FOR PRACTITIONERS.—We invite special attention to the advertisement of the above school, located in St. Louis. It claims to be the first college where no diploma is given to beginners, there being no degrees conferred except upon graduates in medicine.

A PREMIUM worth having is that which is given to every subscriber to that popular monthly magazine, Home, Sweet Home, published by Messrs. Ludden & Bates, Savannah, Ga. It is the Premium Album No. 1, and contains some of the best songs and instrumental pieces ever published; which would cost over $4 if purchased each piece singly. For 50 cents you can get the magazine for a year, and a copy of this Album. If you want interesting home news, subscribe for Home, Sweet Home. Send your name for a free specimen copy te Ludden & Bates' Southern Music House, Savannah, Ga.

Will send the above magazine and the SOUTHERN MEDICAL RECORD one year for $2.25.

SYR. HYPOPHOS COM. C P.—Be sure and examine the advertisement of the above article, as prepared by J. A. McArthur, M.D., and which may be found in the present issue of our journal.

JOURNAL OF THE AMERICAN MEDICAL ASSOCIATION.—The American Medical Association, at its last meeting at Cleveland, Ohio, decided to establish a weekly medical journal, in which to publish the minutes and the papers of that body, instead of in a book of transactions, which was heretofore done.

This journal has, accordingly, been inaugurated under the editorial management of Dr. N. S. Davis, L.L.D., of Chicago. We have received the two numbers first issued, and are pleased to enter this journal on our exchange list. It is a thirty-two page double-column journal, and is creditable in appearance and edited with ability. As a representative journal of the American Medical Profession it will doubtless be warmly received, especially by the more learned and advanced minds in the profession both at home and abroad; and when its able editor shall have time to mature his plans and get fully under headway, we doubt not that we shall have a journal of great and increasing interest. And as the organ through which, from year to year, the great minds of the profession will find expression, it must prove a true and faithful exponent of our medical progress, and of the medical literature of our country.

### *PRACTICAL ANATOMY.*

The bill before the Georgia Legislature to facilitate the study of Practical Anatomy has again failed. That a great State should demand of the Surgeon a knowledge of Anatomy, prosecuting him for mal-practice if he fails to understand it, and yet make penal all efforts to obtain the required knowledge, is an inconsistency unworthy of our boasted civilization.

### *RECEIPTED.*

1882.—Drs. W S Morgan, C J Nichols, Jas Sparks, S S Shropshire. L E Meiggs, T R Simonton.

1883.—Drs. J R Green, E A Speer, P Taylor, Silas Johnson, Jas B Smythe, Rob't Hall, T S Christian, E A Sylar, M M Sherman, Thos A Boggs.

